# Tamoxifen treatment for male breast cancer and risk of thromboembolism: prospective cohort analysis

**DOI:** 10.1038/s41416-018-0369-2

**Published:** 2019-01-17

**Authors:** Holm Eggemann, Anna-Lena Bernreiter, Mattea Reinisch, Sibylle Loibl, Florin-Andrei Taran, Serban-Dan Costa, Atanas Ignatov

**Affiliations:** 1 Department of Obstetrics and Gynaecology, Otto-von-Guericke University, Magdeburg, Germany; 2 Breast Unit, Kliniken Essen-Mitte, Essen, Germany; 3 GBG Forschungs GmbH, Neu-Isenburg, Germany; 4 Department of Obstetrics and Gynaecology, University Hospital Tübingen, Tübingen, Germany; 5 Department of Gynaecology and Obstetrics, University Medical Center, Regensburg, Germany

**Keywords:** Outcomes research, Breast cancer

## Abstract

**Purpose:**

Thromboembolism is a common adverse event in women treated with tamoxifen (TAM) for breast cancer. The risk in male breast cancer patients is poorly investigated. We aimed to examine the risk of thrombotic events after TAM in male breast cancer patients.

**Patients and methods:**

In this prospective cohort study, 448 patients treated between May 2009 and July 2017 for male breast cancer (BC) were assessed for eligibility. Patients with follow-up shorter than 6 months were excluded. The cumulative risk of thromboembolism was evaluated.

**Results:**

The median follow-up was 47 months (range 6–101 months) with a median age of 69.4 years (range 27–89 years). Oestrogen receptor and progesterone receptor expression levels were observed in 98.3 and 94.9% of cases, respectively. During the follow-up period, thrombotic events were documented in 21 (11.9%) of 177 patients receiving TAM and in 1 (2.5%) of 41 patients who did not receive tamoxifen. The estimated incidence was 51.9 per 1000 person-years and 21.5 per 1000 person-years, respectively. Notably, the highest risk was identified in the first 18 months, where 81% of the observed thrombotic events occurred. Patients aged older than 71 years had a significantly increased risk of thrombotic event under TAM treatment than their younger counterparts (*p* = 0.033). History of thrombotic event, cardiovascular and liver disease, as well as additional adjuvant treatment were not associated with increased thrombotic risk.

**Conclusion:**

The risk of thrombotic event in men treated with TAM for breast cancer is markedly increased in the first 18 months of treatment, and should be considered during treatment decisions.

## Introduction

Male breast cancer (BC) is an uncommon disease and its rarity makes the performance of prospective randomised trials very difficult. As a result, the treatment concepts are based on limited retrospective studies and clinical management of the female BC.^1^ Male BC appears to be hormone receptor (HR)-positive in most cases and endocrine therapy is the most important treatment option. In a recent retrospective study of 257 male BC patients, we showed that adjuvant treatment with TAM was associated with a 1.4-fold decreased risk of cancer mortality compared to AI treatment.^2^ Via matching analysis among male and female patients with hormone receptor-positive breast cancer, we demonstrated clearly that the benefit of TAM treatment in male BC is comparable with the effect of TAM in female BC.^3^

One of the most common TAM-associated adverse effects is thromboembolic events.^4,5^ In general, women with BC have an increased risk of thrombotic events compared to women without BC.^6^ However, thus far the adverse effects of TAM in male BC have been poorly investigated. Therefore, identifying the risk profile of TAM in men will help us to further improve the treatment of male BC.

In this large prospective cohort study, the risk of deep-venous thrombosis and thromboembolism in men treated with TAM for BC was investigated. The impact of other risk factors on thrombotic events were also examined.

## Materials and methods

**Fig. 1:**
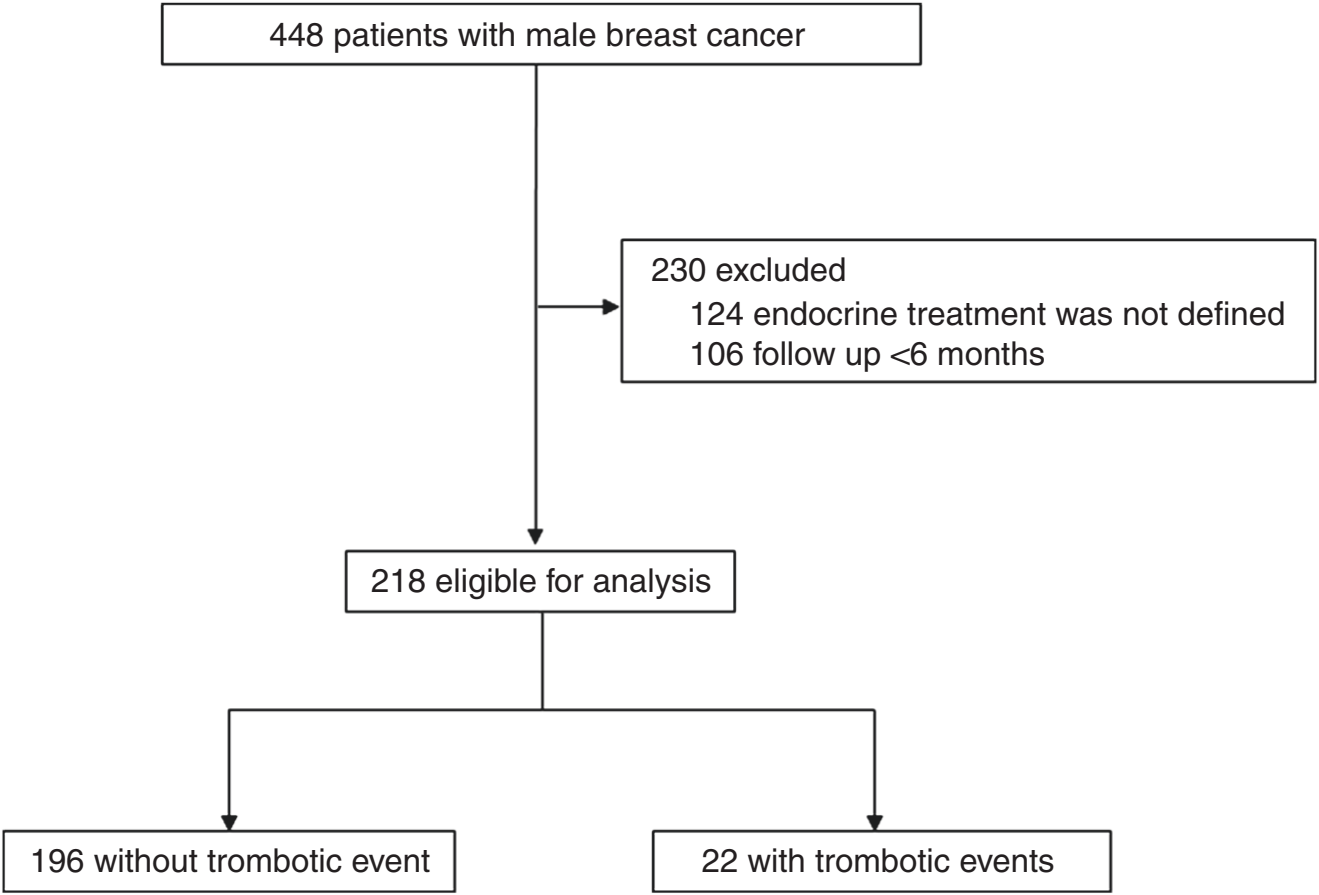
Study design

We investigated cases of male BC from the national prospective cancer registry of Germany. This tumour register contains information about male BC patients: date of diagnosis, patients and tumour characteristics, operative and neo- and/or adjuvant- treatment, date and localisation of relapse, date and cause of death, secondary cancer, comorbidities. We analysed 448 men with primary BC diagnosed between May 2009 and July 2017. We included only patients with non-metastatic invasive HR-positive BC and who had a minimal follow-up of 6 months. Patients were excluded if the endocrine treatment was not defined (*n* = 124) or the follow-up was within 6 months (*n* = 106). Accordingly, of these 448 patients, 218 were eligible for analysis (Fig. 1). The trial was undertaken in accordance with the Declaration of Helsinki and guidelines for Good Clinical Practice and was approved by the Research and Ethical Committee of Otto-von-Guericke University, Magdeburg, Germany. Patients gave written informed consent for data-transfer to the tumour registry before treatment. This trial is registered at the international clinical trial registry platform under the number DRKS00009536 (https://drks-neu.uniklinik-freiburg.de/drks_web/navigate.do?navigationId=trial.HTML&TRIAL_ID=DRKS00009536).

The primary outcome of the study was the rate of thrombotic events under TAM treatment. Thrombotic events were defined as deep-venous thrombosis and/or pulmonary embolism. As a secondary outcome we investigated the influence of other risk factors on thrombotic events in TAM-treated cohort of patients. From each patient the following information was collected: age, year of diagnosis, height and weight, comorbidity, history of thrombotic event, tumour characteristics, tumour treatment strategy, start and stop of TAM treatment, and reason for discontinuation of TAM.

### Statistical analysis

The statistical analyses were performed using SPSS Version 22.0 (SPSS, Chicago, IL, USA). Associations between tumour, patient and treatment characteristics with thrombotic events were analysed by cross-tabulation and tested using the *Χ*^2^ test or Fisher’s exact test. Survival probability was studied using the Kaplan–Meier method. The equality of survival curves was tested using the log-rank test. Cox proportional hazards models were used to assess the influence of adjuvant treatment as an independent prognostic factor and to control further for confounding bias. All tests were two-sided and determined statistically significant if the *p*-value was ≤0.05.

## Results

Between May 2009 and July 2017, 448 men with primary non-metastatic BC were identified and 230 were excluded (Fig. 1). The median follow-up was 47 months (range 6–101 months) and the median age at diagnosis was 69.4 years (range 27–89 years).

In this cohort of patients with male BC, positive estrogenic and progesterone receptor status was observed in 97.7 and 94.0%, respectively. HER2 positivity was demonstrated for 18.1% of the tumours (Table 1). Lymph nodes were involved in 44.1%, lymph vessels in 40.6% and blood vessels in 5.3%. Most of the patients (99.1%) received surgery, 52.4 and 42.6% received radiotherapy and chemotherapy, respectively. Most of the tumours were of intermediate grade of differentiation (62.0%) and had a tumour diameter of < 20 mm (86.3%).

**Table 1:** Clinical and pathological characteristics

Parameter	*N*	%
Median age (years)	70.1 (27–90)	
BMI, kg/m²	27.4 (17–45)	
*Tumor (T) stage*		
0	7	
1	89	3.3
2	71	42.0
3	6	41.0
4	23	2.8
Missing	6	10.8
*Lymph node status*		
Negative	113	
Positive	89	55.9
Missing	16	44.1
*Histological grade*		
1	21	
2	132	9.9
3	60	62.0
Missing	5	28.2
*Blood vessel involvement*		
Negative	178	
Positive	10	94.7
Missing	30	5.3
*Lymph vessel involvement*		
Negative	114	
Positive	78	59.4
Missing	26	40.6
*ER status*		
Negative	5	
Positive	210	2.3
Missing	3	97.7
*PR status*		
Negative	13	
Positive	202	6.0
Missing	3	94.0
*HER2 status*		
Negative	145	
Positive	32	81.9
Missing	41	18.1
*Operative therapy*		
No	2	
Yes	209	0.9
Missing	7	99.1
*Radiotherapy*		
No	100	
Yes	110	47.6
Missing	8	52.4
*Chemotherapy*		
No	124	
Yes	92	57.4
Missing	2	42.6

**Fig. 2:**
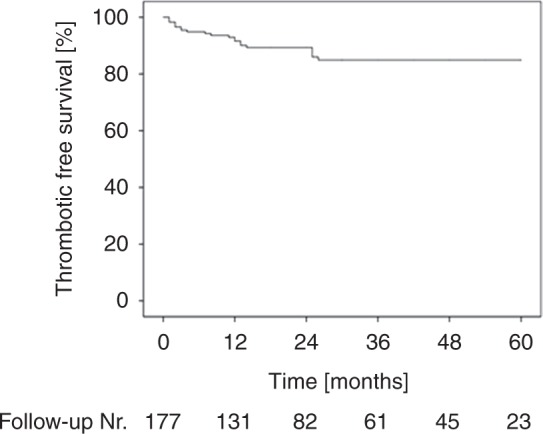
Cumulative incidence of thrombotic events in the first five years of TAM treatment

After further exclusion, 218 men with HR-positive BC were eligible for analysis. Among them, 177 were treated with TAM and 41 received no TAM. In the group of TAM-treated patients 21 (11.9%) experienced a thrombotic event as a side effect. The estimated incidence of thrombotic events in tamoxifen-treated patients was 51.9 of 1000 person-years. Next, we investigated the relationship between the duration of TAM treatment and incidence of thrombotic events. The highest incidence of 6.2% (21.6 per 1000 person-years) was observed in the first year of TAM treatment. In the 2nd and in the 3rdyear 3.4 (13.6 per 1000 person-years) and 2.3% (11.4 per 1000 person-years) of thromboembolism events were observed, respectively. The event-free survival is shown in Fig. 2. During the next 2 years, no additional cases were found. The cumulative incidence curve suggested no further increased risk after the first 3 years of TAM therapy. Thus, the occurrence of thrombosis and embolism had a sharper rise in the first 18 months with a cumulative incidence of 81% of all thrombotic events. In the group of patients who did not received tamoxifen only, 1 (2.5%) thrombotic event was observed in all 41 cases with an incidence of 21.5 of 1000 person-years.

Furthermore, we investigated the influence of other tumour patients and their respective treatment characteristics on thrombotic events. As demonstrated in Table 2, there were no clinical and pathological factors associated with an increased risk of thrombosis in this group of TAM-treated patients. The discontinuation rate of TAM treatment was also examined. TAM was discontinued in 40 (22.6%) of 177 patients (data not shown). Interestingly, in 17 (42.5%) of 40 cases TAM discontinuation was caused by tumour progression and/or relapse. In the remaining 23 (56.5%) patients TAM-induced side effects were the reason for discontinuation.

**Table 2:** Adjusted risk of thromboebolism

Variable	Hazard ratio for events (95% CI)	P value
*Age at diagnosis* *(years)*		
≤70	1.00	0.221
>70	2.32 (0.60–8.98)	
*Tumor (T) size*		
≤20 mm	1.00	0.726
>20 mm	0.68 (0.80–5.82)	
*Histological grade*		
1, 2	1.00	0.650
3	1.28 (0.32–5.09)	
*Lymph node status*		
Negativ	1.00	0.650
Positiv	0.70 (0.15–3.24)	
*HER2 status*		
Negative	1.00	0.782
Positive	0.73 (0.08–6.59)	
*Blood vessel involvement*		
Negative	1.00	0.491
Positive	2.20 (0.23–20.88)	
*Lymph vessel involvement*		
Negative	1.00	0.569
Positive	1.54 (0.35–6.72)	
*Chemotherapy*		
No	1.00	0.375
Yes	0.52 (0.12–2.23)	
*Radiation*		
No	1.00	0.258
Yes	0.46 (0.12–1.77)	

## Discussion

We determined the rate of TAM-induced thrombotic events in patients with male BC, and found that the cumulative risk of thrombosis and embolism is 11.9% and more than 80% of these thrombotic events occurred in the first 18 months after initiation of treatment. The incidence of thrombotic events in tamoxifen-treated patients was 51.9 per 1000 person-years. In the group of patients treated without tamoxifen the thrombotic events were observed in 21.5 per 1000 person-years.

In accordance with previous trials with female BC,^4,7,8^ we have found that TAM is associated with a markedly increased risk of thrombosis. Interestingly, the estimated rate of thrombotic events in our cohort with male BC is somehow higher than the observed rates of thrombosis in women. However, compliant to our data, a retrospective study with male BC thrombotic events were recorded in 6 (9%) out of 64 patients.^9^ In another study with 24 male BC patients, TAM-induced deep-venous thrombosis was observed in 4.2%.^10^ The lower rate of events in the study could be explain by the small number of events (*n* = 1) or by the fact that only deep-venous thrombosis was evaluated, and no information exists about the rate of venous embolism. All this data suggests a slightly elevated risk of thrombosis in men with BC compared to women with BC. Gender-specific risk of thrombotic events has been investigated in numerous trials. Some of them have shown that women exhibit a lower risk of recurrent venous thromboembolism than men, but this data could not be confirmed in other studies.^11^ The reason for this discrepancy is unclear and should be further investigated.

Along with the gender discrepancy, this study reveals another important finding. The incidence of thromboembolism was very high in the first 1.5 years and did not increase after the 3rd year of TAM treatment. Although, this observation is a known phenomenon in women with BC,^6,7,12,13^ our report is the first demonstrating such a steep increase of thrombotic events after initiation of TAM treatment for male BC. In a large population-based study women treated with TAM were at a higher risk for thromboembolism during the first 2 years after initiation of TAM treatment.^7^ Onitilo et al. and Walker et al. have also suggested that TAM-induced thrombotic events occurred early during TAM treatment.^6,12^ The reason for this phenomenon is not well-understood. One explanation is the agonistic activity of TAM on oestrogen receptor^14,15^ and is supported by the finding that hormone replacement therapy on female BC patients is characterised with a similar elevated risk of thrombosis during the initial years of treatment.^16^ Supporting this, an elevated risk of thrombosis has been observed with other antihormonal treatments in patients with prostate cancer.^17^ In general, the effect of hormone therapy on coagulation is multifactorial and includes increased levels/activity of pro-coagulation factors and inhibition of coagulation inhibitors.^18^ An adaptation of the coagulation system to the procoagulant effects of TAM after 1–2 years is proposed. Furthermore, the occurrence of most thrombotic events in the early period of TAM treatment is more likely caused by the influence of conventional risk factors including older age, prolonged immobilisation, extensive surgery, history of stroke and heart disease etc.^4,13^ Notably, in our cohort of patients with male BC only older age was associated with increased risk of thrombotic events. Men older than 70 years were at higher risk of thrombosis than their younger counterparts. However, various other risk factors could not be linked to a further increase of TAM-induced thromboembolism.

This observation is very important with regards to managing the risk of thrombosis. Monitoring using clinical examination and different algorithms should be performed during the period of highest risk.^19^ If possible, TAM should be discontinued if additional risk factors for thromboembolism are present, such as major surgery, prolonged immobilisation, stroke or trauma.^13^ Data from studies comparing female BC and male BC suggest that male BC is largely HR-positive, and the rates of oestrogen and progesterone receptors vary between 70 and 90% indicating that TAM is the most important systemic therapy in male BC.^1,20^ We observed an even higher rate of hormone receptor positivity. Oestrogen and progesterone receptors were positive in 98.3 and 94.9% of the cases, respectively. As we already reported treatment with aromatase inhibitors is inferior to TAM treatment for male BC and the effect of TAM in male BC is comparable with that in female BC.^2,3^ In this context, TAM treatment remains the treatment of choice for HR-positive male BC. In the present study, discontinuation of TAM was observed in 22.6% and is in complete agreement with others.^9,10^ To note, the rate of discontinuation due to TAM-induced side effects was 13%. The remaining discontinuations were due to tumour progression or relapse.

The most important limitation of the present study is the lack of a control group untreated with TAM. It is caused by the small number of patients who did not receive TAM. Thus, we were unable to compare the hazard ratio of TAM with the untreated population and evaluate the influence of additional risk factors on thrombotic risk. Furthermore, we did not evaluate the influence of inherited risk factors on the appearance of thrombotic events in more detail.

However, our study had several important strengths. First, the study is prospective, with a corresponding well-maintained documentation. Second, this is the largest study of adjuvant TAM therapy in male BC patients. Third, this study is population-based, and the exclusion criteria are kept to a minimum resulting in a high level of external validity.

In conclusion, these results will support physicians in treatment decision of patients with male BC and will sensitise them to focus their attention during the first 1–2 years of treatment. In this period an appropriate monitoring of the patients is warranted.
